# Construction and preliminary validation of a tool to measure the needs of adolescents and young adults (AYA) diagnosed with cancer: the QUestionnaire nEEd Cancer AYAs: QUEEC-AYAs

**DOI:** 10.1186/s12955-024-02249-8

**Published:** 2024-04-23

**Authors:** S. Justafré, P. Marino, R. Touzani, F. Dupeyre-Alvarez, P. Dantin, P. Viens, N. Vey, S. Calvin

**Affiliations:** 1 Management Sport Cancer Laboratory UR20122035V, Aix Marseille University and Paoli Calmettes Institute, Marseille, France; 2 Institut Paoli-Calmettes, Aix-Marseille Univ, IRD, SESSTIM, Inserm, Marseille, France; 3 Paoli Calmettes Institute, Marseille, France

**Keywords:** Adolescents and young adults, Cancer, Needs, Patient-reported outcome measures

## Abstract

**Context:**

In France, 2300 adolescents and young adults (AYAs, 15–25 years old) are diagnosed with cancer each year. As soon as the disease is diagnosed, a number of physical, psychological and social needs may arise. The aim of this study is to develop a tool to measure unmet needs that will allow the specificities of AYAs to be understood while allowing health care staff to mobilise the necessary actors to resolve them.

**Methods:**

We developed the Questionnaire nEEd Cancer AYAs (QUEEC-AYAs questionnaire), from two existing questionnaires: the Cancer Needs Questionnaire Young People and the Needs Assessment & Service Bridge. A main sample of 103 AYAs then received and completed the questionnaire in order to conduct an exploratory factor analysis.

**Results:**

The final structure of the QUEEC-AYAs is composed of 7 dimensions and 48 items: information (8 items), cancer care team (6 items), Physical health (4 items), Emotional health (14 items), Sexual & reproductive health (6 items), Health behaviors & wellness (4 items), Daily life (6 items). The questionnaire has a good acceptability and all domains have a Cronbach’s alphas value above 0.80.

**Conclusion:**

The QUEEC-AYAs is the first measure of the psychosocial needs of AYAs available in French. Its systematic use in health care services should improve the coordination of care required by AYAs during and after treatment.

**Trial registration:**

This study was approved by the ethics committee of the Paoli-Calmettes Institute (IRB # IPC 2021-041, 2021 May 20).

**Supplementary Information:**

The online version contains supplementary material available at 10.1186/s12955-024-02249-8.

## Background

It is now well established that a cancer diagnosis has many emotional consequences: shock, disbelief and distress in the face of current events [1, 2]. It gives rise to a number of physical, psychological and social needs in patients that can impact their quality of life [3, 4] after diagnosis and persist beyond the end of treatment [5]. Moreover, to best understand the needs of these patients, the use of patient-reported outcome measures (PROMs, PREMs) is now widely recognised in the literature [6, 7].

Among patients diagnosed with cancer, the adolescent and young adult (AYA) population suffers from a lack of clinical studies and services tailored to their needs. Every year in France, 2,300 young adults are diagnosed with cancer [8]. This young patient population is usually defined by the diagnosis of a tumour in an individual aged between 15 and 25 in France [9], although some international studies extend the upper age to 29 or even 39 [10, 11].

The needs of these patients are mainly assessed by qualitative studies or by the use of validated questionnaires only in adult populations [12]. The CNQ-YP [13] was developed in response to this need for an assessment tool. This is a validated questionnaire for assessing the unmet needs of AYAs during and after treatment. However, to our knowledge, between 2012 and 2022 the CNQ-YP has not been used in international studies due to the complexity of use and the acceptability of the questionnaire by patients [14, 15]. Moreover, the CNQ-YP does not take into account fertility and sexuality dimensions, which are reported as important by AYAs [12, 16] and whose dysfunction linked to the disease or treatment would affect a majority of young people [11]. More recently, the NA-SB [15] questionnaire, derived from the CNQ-YP, questions psychosocial needs more exhaustively but without distinguishing their temporality. The NA-SB was designed as a satisfaction questionnaire and has not been psychometrically validated. In addition, neither scale has been validated in French.

The aim of our study is to use the strengths of these two tools to create a questionnaire for assessing the unmet needs of AYAs in French language.

## Materials and methods

In an iterative process, we translated both questionnaires and presented them to a group of AYAs in order to develop a new tool adapted to French AYAs, combining the strengths of both initial questionnaires. Choices regarding the construction of the tool were based on the literature and qualitative feedback from the focus group AYAs. The questionnaire then underwent a preliminary validation procedure with a sample of AYAs.

### Translation of the CNQ-YP questionnaire

We first translated and back-translated the questionnaire by 2 different translators [17, 18]. Then, a scientific board consisting of a nurse specializing in AYAs, a physician in charge of AYAs, adapted physical activity specialists from the Paoli-Calmettes Institute (IPC - Regional Comprehensive Cancer Centre, Marseille) and researchers from the Management Sport Cancer laboratory drew up a preliminary version of the translated questionnaire.

### Exchanges and focus groups with AYAs

This version of the CNQ-YP was tested on 7 AYAs, aged 18 to 25, in treatment and in remission, recruited at IPC by the nurse coordinator in charge of AYAs, according to the institute’s active file. The completed questionnaires were then sent by the nurse to the research team. The feedback on the questionnaire from the 7 AYAs brought out several aspects regarding, in particular, the excessive length of the questionnaire and the absence of questions about couples, fertility and sexuality.

### Translation of NA-SB questionnaire

In view of the limitations highlighted by the qualitative analysis of the CNQ-YP, the NA-SB questionnaire was translated according to the same methodology and was sent to the same 7 AYAs who had tested the CNQ-YP. This translated version of the NA-SB questionnaire was completed by 3 of them, who reported that it was clearer and shorter, but criticized the loss of timeframes and the binary options which did not make it possible to express their full needs or their intensity.

### Construction of the new scale

We developed the new scale, thanks to the qualitative feedback of AYAs on the CNQ-YP and the NA-SB (Table 1).

**Table 1:** Description of the two questionnaires

	CNQ-YP	NA-SB
**Number of dimensions**	6	9
**Dimensions** ** (Numbers of questions)**	1 Treatment Environment (33) 2 Education (3) 3 Work (3) 4 Information/Activities (5) 5 Feelings/Relationships (14) 6 Daily Life (12)	1 Information (8) 2 Cancer care team (6) 3 Physical health (5) 4 Emotional health (15) 5 Sexual & reproductive health (5) 6 Health behaviors & wellness (7) 7 Work & education (2) 8 Peer support & programming (3) 9 Finances & everyday needs (6)
**Numbers of questions**	70	57
**Consideration of the temporal dimension in questionnaires**	Before treatment During treatment After treatment	None
**Response scale**	Likert 5 points: No need Minimal need Moderate needs Important needs Very important needs	Need help No need help Not applicable

The summary of the items retained from each questionnaire is given in the supplementary table.

The scale proposed in its’ final version is made up of the 57 questions from the NASB, but the response mode and timeframe we taken from the CNQ-YP: The 5-point response scale range from “No need” to “Very high need” and the timeframe allow analysis of patients’ needs both in treatment and in remission, for up to 5 years after their diagnosis [13].

### Participants

Eligible participants were patients aged 18 to 25 at the time of diagnosis; having been diagnosed with cancer within the last 5 years. People unable to give their consent, not having a basic understanding of the French language, not having participated in the pilot study or not being affiliated with social security or equivalent were not included.

### Data collection

To facilitate delivery and response, the questionnaire was digitized and placed on a secure platform to enable the AYAs to respond directly online. A link was generated and emailed by the nurse to all the AYAs at IPC who met the inclusion criteria. Before completing the questionnaire, the AYAs were invited to answer some socio-demographic questions (sex, age, medical situation, socio-occupational situation, personal situation). Systematic reminder emails were sent 2 weeks, 1 month and 1.5 months after the first contact. Returning the online questionnaire was considered as providing written informed consent to participate.

### Statistical analysis

Patients’ characteristics were reported using usual descriptive statistics (frequencies, means and proportions). Scale items for which > 70% of participants indicated no or low needs were removed [19–21]. The factor structure of the questionnaire was assessed using exploratory factor analysis (EFA). Extracted factors were orthogonally rotated using the Varimax procedure (Eigenvalue > 1 criteria). Items were included on a factor if they displayed a loading of at least 0.4 on one of the factors [22, 23]. If an item loaded across several factors with similar loadings, it was attributed to the initial factor structure. When redundancy between items was observed (Pearson correlation coefficient *r* ≥ 0.70), the item retained was the one with the higher loading. A second EFA was carried out after deleting the items correlated and those with low loading [24]. In cases where the loading was less than 0.4, the item was retained if > 50% of participants indicated a high or very high need for the item. Sampling adequacy was assessed measuring the Kaiser-Meyer-Olkin (KMO) [25] test and Bartlett’s test of sphericity. A KMO value over 0.5 and a significance level for Bartlett’s test (*p* < 0.05) suggested there was correlation in the data.

Internal consistency was measured by calculating the Cronbach’s alpha coefficient for each dimension of the scales. A Cronbach’s alpha coefficient ≥ 0.7 was considered satisfactory [26].

For each subscale as well as the global scale, a score was generated by summing all responses (from 0 (no need) to 4 (very high need)). A higher score indicated a higher level of need.

All analyses were performed using STATA version 15 (StataCorp, College Station, TX, USA).

## Results

### Participants

522 AYAs meeting the inclusion criteria were contacted by the nurse responsible for AYAs at IPC and were emailed the link to the questionnaire. After 3 reminder emails sent by the nurse, 103 AYAs completed the questionnaire. The socio-demographic characteristics of the respondents are summarized in Table 2.

**Table 2:** Characteristics of the 103 patients

Sex	
Female	65 (63.1%)
Male	38 (36.9%)
**Age**	
18–20	5 (4.9%)
20–25	43 (41.7%)
> 26	55 (53.4%)
**Management phase**	
In treatment	21 (20.4%)
≤ 2 years remission	46 (44.7%)
> In remission + 2 years	36 (35%)
**Socio-occupational status**	
Employed	51 (49.5%)
Student	24 (23.3%)
Inactive	28 (27.2%)
**Personal situation**	
Single	45 (44.1%)
In couple	57 (55.9%)

### Exploratory factor analysis (EFA)

Of the 103 participants completing the questionnaire, 96 had no missing values for any items. No items had > 90% of participants reporting the same level of need, indicating reasonable variability of responses within items. Missing data ranged from 0 to 2.91% depending on the items.

### First exploratory factor analysis (57-item questionnaire)

In its initial version, the questionnaire included 57 items. We first conducted an EFA on this 57-item version. Following this first analysis, we deleted the dimension “Work and education” consisting of one item related to education needs “I wanted more help with managing my school life while going through cancer treatment” and one item related to work needs “I wanted more help with managing my working life while going through cancer treatment” because for both items loadings were below 0.4. From the 55-remaining items, 5 items were removed because more than 70% of the patients declared no or minimal needs for these 5 items. These items were: “I wanted more help with managing loss of walking ability; I wanted more information about smoking; I wanted more information about drug or alcohol use; I wanted more help with having childcare during my cancer care appointments; I wanted more help with having stable housing”. Assessment of the inter-item Spearman correlation matrix between items revealed that two items “I wanted more help with being able to spend time with people my own age / I wanted more help with participating in social activities”, had correlations > 0.90 as well as two items “I wanted more help with worrying about my cancer spreading / I wanted more help with worrying about my cancer returning or getting another type of cancer”. The items selected in the scale were those for which the loading was higher.

### Second exploratory factor analysis (revised 48-item questionnaire)

Based on the results of the first analysis, we conducted a second EFA on the revised 48-item questionnaire. We found a factor structure with 7 factors. The items and factor loadings corresponding to the 7 extracted factors are presented in Table 3.

**Table 3:** Factor structure of the questionnaire from the revised factor analysis (48-item questionnaire)

INFORMATION			Factor1	Factor2	Factor3	Factor4	Factor5	Factor6	Factor7
**1**	*I wanted more information about*:	*My cancer diagnosis*		**0.705**					
**2**		*The short-term side effects of treatment*		**0.830**					
**3**		*The long-term side effects of treatment*		**0.784**					
**4**		*What will happen when treatment finishes*		**0.720**					
**5**		*My disease status*		**0.763**					
**6**		*My test results*		**0.795**					
**7**		*What to do if I have side effects from my treatment*		**0.777**					
**8**		*How my genetics may or may not have impacted my diagnosis and treatment*		0.349					
**CANCER CARE TEAM**									
**9**	*I felt the need for my cancer treatment team to*:	*Respect me as an individual, not just a cancer patient*			**0.736**				
**10**		*Offer to talk to me in private, without my family or friends*			**0.520**				
**11**		*Explain what they were doing in a way I can understand*			**0.745**				
**12**		*Encourage me to ask questions*			**0.770**				
**13**		*Engage me in decision-making about my treatment and respect my decisions*			**0.674**				
**14**		*Ask me about my treatment concerns*			**0.634**				
**PHYSICAL HEALTH**									
**15**	*I wanted more help with*:	*Managing pain*						**0.640**	
**16**		*Managing my medications*						**0.694**	
**17**		*Managing physical side effects of treatment*						**0.661**	
**18**		*Managing feeling tired / fatigued*						**0.601**	
**EMOTIONAL HEALTH**									
**19**	*I wanted more help with*:	*Feeling anxious or scared*	**0.586**						
**20**		*Feeling depressed*	**0.620**						
**21**		*Worrying about my cancer returning or getting another type of cancer*				**0.424**			
**22**		*Worrying about how my family is coping*	**0.495**						
**23**		*Coping with changes in my dating or romantic life*	**0.454**						
**24**		*Coping with changes in my relationships with my family members*	**0.675**						
**25**		*Coping with changes in my relationships with friends*	**0.725**						
**26**		*Feeling independent*	**0.789**						
**27**		*Coping with changes in my physical ability*	**0.667**						
**28**		*Coping with changes in my appearance*	**0.666**						
**29**		*Coping with not being able to do the same things as other people my age*	**0.619**						
**30**		*Managing the emotional side effects of treatment*	**0.607**						
**31**		*Being able to make plans or think about the future*	**0.763**						
**32**		*Entre capable d’avoir des projets ou de penser à l’avenir*	**0.643**						
**SEXUAL & REPRODUCTIVE HEALTH**									
**33**	*I wanted more information about*:	*My risks of infertility and my solutions to preserve my fertility*				**0.683**			
**34**		*Infertility treatment and other solutions for having children later (e.g. sperm/egg freezing, in vitro fertilization, etc.)*				**0.730**			
**35**		*Sexuality and intimacy during cancer treatment*				**0.750**			
**36**		*Sexual side effects of treatment (e.g. sexual dysfunction)*				**0.732**			
**37**		*The effects of treatment on long-term hormonal changes*				**0.636**			
**HEALTH BEHAVIORS & WELLNESS**									
**38**	*I wanted more information about*:	*Nutrition*					**0.725**		
**39**		*Exercise or physical activity*					**0.691**		
**40**		*Getting enough or better-quality sleep*					**0.667**		
**41**		*Spiritual support or resources*					**0.496**		
**42**		*Alternative therapies (herbal treatment, acupuncture, massage therapy, meditation, etc.)*	**0.519**						
**DAILY LIFE**									
**43**	*I wanted more help with*:	*Being able to spend time with people my own age*							**0.631**
**44**		*Being able to talk to people my own age who have been through a similar cancer treatment experience*							**0.543**
**45**		*Paying my bills*							**0.536**
**46**		*Scholarship or loan repayment options*							**0.673**
**47**		*My health insurance (e.g., access/eligibility, coverage, cost)*							**0.426**
**48**		*Getting to and from my cancer care appointments*							**0.480**

Bartlett’s test of sphericity was significant (χ2 = 3541.57; *p* < 0.001) and the KMO test for sampling adequacy (KMO = 0.78) indicated a medium fit for factor analysis.

All 48 items except one (Q1-8) had loadings > 0.4. We decided, however, to retain Q1-8 even with a loading = 0.349 because for this item 54% of patients reported high or very high needs.

10 items did not have unique factor loadings > 0.4. For all of these 10 items, we retained the item in the factor with the highest loading. The factor loading for item 21 (Table 3) initially grouped in the subscale “Emotional health*”* was much higher for the subscale “Sexual and reproductive health”. Similarly, item 42 has moved from the factor “Health and wellness” to the factor “Emotional health”.

### Internal consistency

The Cronbach’s alpha reliability coefficients ranged from 0.81 to 0.94, which indicates a very good internal consistency of each subscale (Table 4).

**Table 4:** Score, distribution, and cronbach’s alpha of the QUEEC-AYAs scale

Factor	No. of items	Range	Range observed	N	Median	Mean	SD	% score min	% score max	Alpha
1	14	0–56	0–56	100	26	27.04	14.87	3	3	0.93
2	8	0–32	1–28	101	22	21.33	5.72	0.99	11.88	0.9
**3**	**6**	**0–24**	**1–24**	**101**	**17**	**15.9**	**6.05**	**0.99**	**7.92**	**0.89**
**4**	**6**	**0–24**	**6–24**	**101**	**12**	**11.97**	**7.26**	**5.94**	**5.94**	**0.89**
**5**	**4**	**0–16**	**0–16**	**101**	**6**	**6.55**	**4.43**	**8.91**	**4.95**	**0.79**
**6**	**4**	**0–16**	**0–16**	**100**	**7**	**6.92**	**4.61**	**14**	**7**	**0.84**
**7**	**6**	**0–24**	**0–24**	**100**	**7**	**8.01**	**6.08**	**12**	**2**	**0.79**
**Total**	**48**	**0–192**	**2–188**	**96**	**98.5**	**96.45**	**36.29**	**1.04**	**2.08**	**0.95**
	KMO = 0.78 mean adjustment									

For each dimension, sub-scores were calculated as the sum of response to items (with 0 = no need, 1 = low need, 2 = moderate need, 3 = high need, 4 = very high need)

### Scoring of the scale, floor and ceiling effects

Descriptive statistics of the subscales and the global scale scores are presented in Table 4. Of the 103 participants completing the questionnaire, a total of 96 responded to all 48 items (no missing values). The average total score was 96.45 (SD = 36.29); it ranged from 2 to 188 (maximum possible score = 192). The proportions of participants who scored the minimum and maximum scores for each factor are also detailed in Table 4. No ceiling and floor effects were observed. The minimum score from each subscale ranged from 0.99 to 14% of patients, while the maximum score ranged from 2 to 11.88% of patients.

## Discussion

This study aims to establish a scale to measure the unmet needs of AYAs diagnosed with cancer and to evaluate its psychometric properties. The scale was constructed from existing tools: the CNQ-YP [13] and the NA-SB [15]. The CNQ is a validated questionnaire covering a broad timeframe for evaluation of needs during and after treatment, but does not include the sexual dimension of needs. This dimension is, however, reported as important by AYAs [12, 27]. The NA-SB, derived from the CNQ-YP, asks more exhaustively about needs but without distinguishing their timeframe; it has not been psychometrically validated. Moreover, neither of the 2 scales has been validated in French.

The initial version of our questionnaire is very close to the structure of the NA-SB with the same number of questions. The headings, the timeframes and the form of the responses were, however, adapted on the basis of the CNQ-YP.

The final version of the questionnaire has 7 dimensions with 48 items: Information (8 items), Cancer Care Team (6 items), Physical Health (4 items), Psychological Health (14 items), Sexual and Reproductive Health (6 items), Health Behaviours and Wellness (4 items) and Daily Life (6 items).

The “Work and education” dimension of the NA-SB was not retained because, for each of the 2 items it contains, the load was much smaller than the chosen criterion (> 0.4), with even a negative loading. This is explained by the fact that the dimension contains only two items, which are moreover mutually exclusive (a person in education is not in work). The poor factor loading could also be explained by the fact that 27.2% of the survivors were inactive, and 20.4% were actually receiving active treatment. In the context where these dimensions appear to be very important to survivors [28], we recommend the development of a module adapted so as to explore these two dimensions.

### Adjustments induced by the floor effects

The questions on “information about smoking and alcohol” show respectively 76% and 83% absence of needs and were therefore removed from the final questionnaire. These high results are probably due to the “feeling of being informed” found among 15 to 30-year-olds [29]. The impact of awareness-raising campaigns in the hospital and more generally by the public authorities contributes to the diffusion of information on these topics [30].

Likewise, the question on “loss of walking ability” gives 72% “No need” or “Low need” answers, suggesting that this symptom is very infrequent in the treatment phase and is not a consequence of the treatments. This item was therefore also removed from the final version of the questionnaire.

Likewise, the questions on “Help with housing” and “Help with childcare” had respectively 83% and 87% “No need” or “Low need” answers and were removed. It is important to note that, in France, one in two 18 to 29-year-olds still live with their parents [31] and in 2022 the average age of giving birth was 31 [32], which probably explains the lack of needs declared by this AYA population.

### Adjustments induced by the loadings

We observed that the items “Worrying about my cancer spreading” and “Worrying about my cancer returning” were strongly correlated, and therefore supplied redundant information. The distinction between recurrence and spread may indeed be vague for patients. We therefore retained only one of the two items in the final questionnaire, the one with the greater loading. Likewise, the item “Participating in social activities” was strongly correlated with “Being able to spend time with people my own age”, which is explained by the fact that social activities in the AYA population are often done with people of the same age. We again retained only one item, the one with the higher loading in the factor analysis.

Finally, the item “How my genetics may or may not have impacted my diagnosis and treatment” in the dimension “Information” was retained despite its weak loading in the exploratory factor analysis. Despite a loading of < 0.4, we observe a high rate of needs expressed (54% high or very high need), which suggests that the question of the genetic aspects of the disease is a matter of concern for AYAs and deserves to be retained in the questionnaire.

### Adjustments induced by the exploratory factor analysis

Finally, some items were moved to another dimension after the EFA. Thus, the question on “Finding information on alternative therapies (herbal treatment, acupuncture, massage therapy, meditation, etc.)”, initially contained in the category “Health and wellness” was moved to the “Emotional health” dimension because of a greater loading in the latter. This shift seemed to belong to a logic of provision of these alternative treatments in the treatment of the psychological consequences of a cancer [33].

Similarly, the question on “Having what I need to cope with my diagnosis”, initially placed in the “Psychological consequences” was moved to the dimension “Sexual and reproductive health”. Including this item in the “Sexual health” dimension would make it possible to evaluate the need bring in specific support to meet these needs. It will also make it possible to refine understanding of this dimension which is recurrently mentioned by patients in the literature.

### Acceptability of the questionnaire

In our study, the rate of response to the questionnaire (number of questionnaires returned / number of questionnaires distributed) is 17.85%. This is low relative to the rates commonly reported in the literature– between 40% and 50%– in a general way and relative to those for the validation of the CNQ-YP [13]. This can be explained by the difference in how the questionnaire was distributed. For the validation of the CNQ-YP, 577 AYAs were identified and contacted, first with a preliminary request for consent, then with the questionnaire and the material for answering it. The rate of response was therefore calculated by reference to the number of patients having returned the consent form.

In our study, the link to the online questionnaire completion was sent by email and returning the questionnaire was considered as giving informed consent. Our rate of response was therefore based on the number of patients contacted and not on the number of patients giving consent, which explains our lower response rate.

However, it is important to note that 96 responses out of 103 had no missing data, which suggests that the questions are clear and easy to understand and that the questionnaire is easy to complete. This good acceptability of the questionnaire needs to be confirmed for the modified final version in a later confirmatory study.

### Limitations of the study

The first limitation lies in the fact that our study was carried out in a single cancer center, which only admits AYAs aged 18 and over, thus excluding AYAs aged 15 to 18. However, insofar as the literature points to similar needs whatever the age, particularly with regard to relational and psychological aspects [34] and sexual aspect [35], our questionnaire could be a relevant tool for assessing the needs of young people aged 15 to 18.

Secondly, we were not able to conduct a test-retest analysis. The difficulties of recruiting from the population of AYAs [36, 37] led us to choose a method for collection of consent/questionnaire/responses that minimizes the loss of respondents, in a population that is not very captive. For this reason, we did not ask AYAs to return the consent/questionnaire to the nurse coordinator, which would have made it possible to associate an inclusion number, and ensure longitudinal follow-up. We thought this supplementary step might have decreased the response rate to our study and we have chosen a direct anonymous online response mode which in turn prevents a retest.

Finally, this study has a restricted sample of respondents. Only 103 patients completed the questionnaire out of 577 contacted by email. Other studies moving toward development of measuring tools for this population have obtained similar results in their recruitment [13, 38, 39].

### Practical applications

The present questionnaire is a multidimensional measure of unmet needs adapted to the specificities of French AYAs (aged 18–30). The structure of this tool was designed so as to facilitate its everyday use in the AYA care departments and help to improve care coordination. Easier and more systematic evaluation of patients’ needs by the caring departments throughout the care pathway should allow more clearly identified referral to adapted support and best meet the patients’ needs. The questionnaire is also designed to evaluate patients’ information needs on sexual aspects and so fills a gap noted in the literature [40]. With improved understanding of the information that patients lack, the care professionals will be able to target the information to be provided and so prevent certain aberrations, particularly with regard to the exclusive use of non-conventional treatments [41].

## Conclusion

This study is a preliminary validation of a need-measuring tool validated in French, that is usable both by researchers and by care teams and whose psychometric properties allow reliable measurement of unmet needs during and after cancer treatment. Psychometric properties will however have to be confirmed in future prospective studies using confirmatory factor analysis.

### Electronic supplementary material

Below is the link to the electronic supplementary material.

Supplementary Material 1

Supplementary Material 2

## Data Availability

The data is available on request from the corresponding author: S. Justafré sebastien.justafre@univ-amu.fr.
