# Supplementary material for: Construction and preliminary validation of a tool to measure the needs of adolescents and young adults (AYA) diagnosed with cancer: the QUestionnaire nEEd Cancer AYAs: QUEEC-AYAs
Source: Health Qual Life Outcomes. 2024 Apr 23;22:36. doi: 10.1186/s12955-024-02249-8 (PMC11036591; doi:10.1186/s12955-024-02249-8)
Supplement: Supplementary file 2 — Supplementary Material 2 [file 12955_2024_2249_MOESM2_ESM.pdf]

Mesure des besoins des AJAs

Nous essayons de trouver de meilleures façons d'aider les adolescents et jeunes adultes ayant été atteints d'un cancer. Pour ce faire, nous souhaitons vous interroger sur les besoins que vous avez éprouvés sur le plan physique, psychologique et social depuis le diagnostic de votre cancer. Répondez à chaque question en choisissant la réponse qui correspond le mieux à votre niveau de besoins. Cinq réponses sont proposées.

Aucun besoin	On a satisfait tous mes besoins pour ce problème ou ce problème n'en a pas été un pour moi.
Besoins minimes	J'avais besoin de très peu d'aide pour faire face à ce problème mais je n'en ai pas obtenu.
Besoins modérés	J'avais modérément besoin d'aide pour faire face à ce problème mais je n'en ai pas obtenu.
Besoins importants	J'avais fortement besoin d'aide pour faire face à ce problème mais je n'en ai pas obtenu.
Besoins très importants	J'avais très fortement besoin d'aide face à ce problème mais je n'en ai pas obtenu.

Conformément aux dispositions en vigueur du RGPD, les informations que vous nous confiez seront traitées de manière strictement confidentielle : elles seront analysées statistiquement et votre anonymat sera parfaitement respecté.

Nous vous remercions du temps que vous allez consacrer à ce questionnaire.

**CADRE DE L'ANNONCE**							
**Avant le traitement**			**Aucun besoin**	**Besoins minimes**	**Besoins modérés**	**Besoins importants**	**Besoins très importants**
**1**	***j'ai éprouvé les besoins d'informations concernant***	**Le diagnostic de mon cancer**					
**2**	***j'ai éprouvé les besoins d'informations concernant***	**Les effets secondaires du traitement ont couru terme**					
**3**	***j'ai éprouvé les besoins d'informations concernant***	**Les effets secondaires du traitement à long terme**					
**4**	***j'ai éprouvé les besoins d'informations concernant***	**Ce qui se passera à la fin du traitement**					
**5**	***j'ai éprouvé les besoins d'informations concernant***	***L'état de ma maladie***					
**6**	***j'ai éprouvé les besoins d'informations concernant***	**Mes résultats d'examen**					
**7**	***j'ai éprouvé les besoins d'informations concernant***	**Ce que je dois faire si j'ai des effets secondaires dus au traitement**					
**8**	***j'ai éprouvé les besoins d'informations concernant***	***La façon dont les facteurs génétiques peuvent ou non avoir influencé mon diagnostic et mon traitement***					
**PERSONNEL PRENANT EN CHARGE MON CANCER**							
**Pendant le traitement**			**Aucun besoin**	**Besoins minimes**	**Besoins modérés**	**Besoins importants**	**Besoins très importants**
**9**	***j'ai éprouvé les besoins que les professionnels présents***	**Me respecte en tant que personne, pas seulement en tant que patient(e) atteint d'un cancer**					
**10**	***j'ai éprouvé les besoins que les professionnels présents***	**Me propose des discussions en privé, sans ma famille ou mes amis**					
**11**	***j'ai éprouvé les besoins que les professionnels présents***	**M'explique ce qu'il fait dans un langage que je peux comprendre**					
**12**	***j'ai éprouvé les besoins que les professionnels présents***	**M'encourage à poser des questions**					
**13**	***j'ai éprouvé les besoins que les professionnels présents***	**Me font participer à la prise des décisions concernant mon traitement et respecter mes décisions**					
**14**	***j'ai éprouvé les besoins que les professionnels présents***	***Me demande si j'ai des préoccupations concernant mon traitement***					

CONSÉQUENCES PHYSIQUES LIÉES AU CANCER							
Au cours du dernier mois			Aucun besoin	Besoins minimes	Besoins modérés	Besoins importants	Besoins très importants
15	*j'ai éprouvé le besoin d'être mieux aidé(e) pour gérer*	La douleur					
16	*j'ai éprouvé le besoin d'être mieux aidé(e) pour gérer*	Gérer mes médicaments					
17	*j'ai éprouvé le besoin d'être mieux aidé(e) pour gérer*	Les effets secondaires physiques du traitement					
18	*j'ai éprouvé le besoin d'être mieux aidé(e) pour gérer*	Une sensation de fatigue ou d'épuisement					

CONSÉQUENCES PSYCHOLOGIQUES LIÉES AU CANCER							
Au cours du dernier mois			Aucun besoin	Besoins minimes	Besoins modérés	Besoins importants	Besoins très importants
19	*j'ai éprouvé le besoin d'être mieux aidé(e) pour*	Gérer l'anxiété ou la peur					
20	*j'ai éprouvé le besoin d'être mieux aidé(e) pour*	Gérer la tristesse					
21	*j'ai éprouvé le besoin d'être mieux aidé(e) pour*	Gérer ma crainte que mon cancer récidive ou que j'aie un autre cancer					
22	*j'ai éprouvé le besoin d'être mieux aidé(e) pour*	Gérer ma crainte que ma famille ait du mal à affronter cette épreuve					
23	*j'ai éprouvé le besoin d'être mieux aidé(e) pour*	Faire face aux changements dans ma vie sentimentale ou dans mes rencontres amoureuses					
24	*j'ai éprouvé le besoin d'être mieux aidé(e) pour*	Faire face aux changements dans ma relation avec les membres de ma famille					
25	*j'ai éprouvé le besoin d'être mieux aidé(e) pour*	Faire face aux changements dans ma relation avec mes amis					
26	*j'ai éprouvé le besoin d'être mieux aidé(e) pour*	Me sentir indépendant(e)					
27	*j'ai éprouvé le besoin d'être mieux aidé(e) pour*	Faire face à l'évolution de mes capacités physiques					
28	*j'ai éprouvé le besoin d'être mieux aidé(e) pour*	Faire face à l'évolution de mon apparence					

29	*j'ai éprouvé le besoin d'être mieux aidé(e) pour*	**Vivre avec le fait que je ne peux pas faire les mêmes choses que les personnes de mon âge**					
30	*j'ai éprouvé le besoin d'être mieux aidé(e) pour*	***Gérer les effets secondaires émotionnels du traitement***					
31	*j'ai éprouvé le besoin d'être mieux aidé(e) pour*	**Entre capable d'avoir des projets ou de penser à l'avenir**					
32	*j'ai éprouvé le besoin d'avoir plus d'informations concernant*	**D'autres traitements non conventionnels (traitement à base de plantes, acupuncture, massothérapie, méditation, etc.)**					
**SANTÉ SEXUELLE ET REPRODUCTIVE**							
**Au cours du dernier mois**			**Aucun besoin**	**Besoins minimes**	**Besoins modérés**	**Besoins importants**	**Besoins très importants**
33	*j'ai éprouvé le besoin d'avoir plus d'informations concernant*	***Mes risques d'infertilité et mes solutions pour préserver ma fertilité***					
34	*j'ai éprouvé le besoin d'avoir plus d'informations concernant*	***Le traitement de l'infertilité et les autres solutions pour avoir des enfants plus tard (p. ex. congélation des spermatozoïdes/ovocytes, fertilisation in vitro, etc.)***					
35	*j'ai éprouvé le besoin d'avoir plus d'informations concernant*	***La sexualité et l'intimité pendant le traitement du cancer***					
36	*j'ai éprouvé le besoin d'avoir plus d'informations concernant*	***Les effets secondaires du traitement sur le plan sexuel (p.ex. dysfonction sexuelle)***					
37	*j'ai éprouvé le besoin d'avoir plus d'informations concernant*	***Les effets du traitement sur les changements hormonaux à long terme***					
38	*j'ai éprouvé le besoin d'être mieux aidé(e) pour*	**Répondre à mes besoins pour vivre avec mon diagnostic**					
**COMPORTEMENTS FAVORISANT LA SANTÉ ET LE BIEN ÊTRE**							
**Au cours du dernier mois**			**Aucun besoin**	**Besoins minimes**	**Besoins modérés**	**Besoins importants**	**Besoins très importants**
39	*j'ai éprouvé le besoin d'avoir plus d'informations concernant*	***La nutrition à adopter***					
40	*j'ai éprouvé le besoin d'avoir plus d'informations concernant*	***La pratique de sport ou d'activité physique***					

**41**	***j'ai éprouvé le besoin d'avoir plus d'informations concernant***	***Le nombre d'heures de sommeil ou la qualité du sommeil***					
**42**	***j'ai éprouvé le besoin d'avoir plus d'informations concernant***	***Un soutien ou des ressources d'ordre spirituel***					
**VIE QUOTIDIENNE**							
**Au cours du dernier mois**			**Aucun besoin**	**Besoins minimes**	**Besoins modérés**	**Besoins importants**	**Besoins très importants**
**43**	***j'ai éprouvé le besoin d'être mieux aidé(e) pour***	**Pouvoir passer du temps avec des personnes de mon âge**					
**44**	***j'ai éprouvé le besoin d'être mieux aidé(e) pour***	**Pouvoir discuter avec des personnes de mon âge qui ont reçu un traitement contre le cancer similaire**					
**45**	***j'ai éprouvé le besoin d'être mieux aidé(e) pour***	***Payer mes factures***					
**46**	***j'ai éprouvé le besoin d'être mieux aidé(e) pour***	***Gérer les possibilités de bourses d'études ou de remboursement de prêts***					
**47**	***j'ai éprouvé le besoin d'être mieux aidé(e) pour***	***Mon assurance santé (p.ex., pour l'accès/les conditions à remplir, la couverture, le coût)***					
**48**	***j'ai éprouvé le besoin d'être mieux aidé(e) pour***	***Les déplacements pour mes séances de traitement du cancer***					
